# Insulin-induced lipid body accumulation is accompanied by lipid remodelling in model mast cells

**DOI:** 10.1080/21623945.2019.1636624

**Published:** 2019-07-16

**Authors:** Johnny T. Aldan, Chad Jansen, Mark Speck, Kristina Maaetoft-Udsen, Edward A. Cordasco, Mata’Uitafa Faiai, Lori M.N. Shimoda, William E. Greineisen, Helen Turner, Alexander J. Stokes

**Affiliations:** a Laboratory of Immunology and Signal Transduction, Chaminade University, Honolulu, HI, USA; b Laboratory of Experimental Medicine, John A. Burns School of Medicine, University of Hawai‘i, Honolulu, HI, USA

**Keywords:** Cardiolipin, arachidonic acid, mast cells, lipidome

## Abstract

Mast cell lipid bodies are key to initiation, maintenance and resolution of inflammatory responses in tissue. Mast cell lines, primary bone marrow-derived mast cells and peripheral blood basophils present a ‘steatotic’ phenotype in response to chronic insulin exposure, where cells become loaded with lipid bodies. Here we show this state is associated with reduced histamine release, but increased capacity to release bioactive lipids. We describe the overall lipid phenotype of mast cells in this insulin-induced steatotic state and the consequences for critical cellular lipid classes involved in stages of inflammation. We show significant insulin-induced shifts in specific lipid classes, especially arachidonic acid derivatives, MUFA and PUFA, the EPA/DHA ratio, and in cardiolipins, especially those conjugated to certain DHA and EPAs. Functionally, insulin exposure markedly alters the FcϵRI-induced release of Series 4 leukotriene LTC4, Series 2 prostaglandin PGD2, Resolvin-D1, Resolvin-D2 and Resolvin-1, reflecting the expanded precursor pools and impact on both the pro-inflammation and pro-resolution bioactive lipids that are released during mast cell activation. Chronic hyperinsulinemia is a feature of obesity and progression to Type 2 Diabetes, these data suggest that mast cell release of key lipid mediators is altered in patients with metabolic syndrome.

## Introduction

Metabolic syndrome is associated with a chronic state of hyperinsulinemia, prior to the onset of Type 2 Diabetes mellitus [1,2]. Chronic insulin elevation has functional consequences for numerous cells, tissues and organs in the body, including those of the immune system [3–5]. We have previously shown that chronic insulin elevation alters mast cell functional phenotypes *in vitro*, and there is *in vivo* and clinical evidence that altered levels of insulin affect the outcomes of allergic and anaphylactic inflammatory responses [6–9]. Chronic insulin exposure quantitatively alters the lipid content of mast cells, causes a steatosis-like accumulation of lipid bodies similar to that observed in neutrophils and macrophages under conditions of infection. However, qualitative changes in the cellular profiles of bioactive lipids and their precursors have not been extensively explored. This is an important question arising from the marked effects that lipid remodelling has upon the net pro-and anti-inflammatory capacity of cells such as mast cells [10–14].

Most lipid mediators that been shown to regulate inflammation are derived from omega-6 (n-6) or omega-3 (n-3) fatty acids [10,14–16]. These mediators include arachidonic acid (AA; 20:4n-6), linoleic acid (LA; 18:2n-6), eicosapentaenoic acid (EPA; 20:5n-3), and docosahexaenoic acid (DHA; 22:6n-3). Oxidation catalyzed by cyclooxygenases, lipoxygenases, or cytochrome P450 forms the bioactive metabolite from these precursors. Acute changes in cellular status have been shown to remodel key lipid populations in mast cells and other immune cells, changing the outcome of mast cell activation and in some cases switching the cell between a pro-inflammation and a pro-resolution phenotype [7,16–20].

Several studies have evaluated the location of the bioactive lipid precursor AA in mast cells, evaluating distribution between membrane phospholipid (phosphatidylcholine (PC), phosphatidylethanolamine (PE) and phosphatidylserine (PS)) and free fatty acid (FFA) forms of AA. The distribution within these pools changes in response to mast cell activation in response to both calcium ionophore and FcϵRI stimulation [19,21,22]. These changes are functionally important as the location of the AA changes its proximity and availability to phospholipases that are concomitantly activated and catalyse pathways leading to synthesis of prostaglandins, leukotrienes, thromboxanes, HETEs, resolvins and endocannabinoids [13,23,24]. Similarly, functional importance is ascribed to the ratios between omega-3 and omega-6-fatty acids and the abundance of DHA and EPA pools. These are precursors for inflammation resolving factors (protectins, resolvins, and maresins). Since n-6 and n-3 fatty acids are generally regarded as pro-inflammatory, and anti-inflammatory, respectively, the cellular abundance of these forms in mast cells has consequences for tissue inflammatory responses [25,26].

In the context of metabolic syndrome and obesity, lipid remodelling has been studied in adipose tissue and adipocytes [20]. In addition to the formation and storage of large lipid droplets, adipose depot expansion and increased infiltration of pro-inflammatory cells such as macrophages, obesity is also linked to qualitative changes in adipocyte lipid content. In particular, switching towards a more pro-inflammatory lipid profile (increased ratio of poly-unsaturated fatty acids (PUFA) to mono-unsaturated fatty acids (MUFA), altered ratio of DHA and EPA, altered AA levels. In addition, infiltrating macrophages remodel their lipid precursor pools when in obese adipose tissue contexts. However, the effect of an altered metabolic/glycemic environment upon mast cell lipid profiles has not been studied.

In prior studies we have shown that chronic insulin exposure is associated with elevated lipid body numbers, overall increases in cellular lipid content and elevated leukotriene C4 (LTC4) production in model mast cells [6–9,27]. In the current study, we use a similar model system in which a mucosal mast cell-like line is exposed to elevated insulin over a chronic time period. Our data suggest remodelling of the mast cell lipidome and altered functional release of bioactive lipids in response to chronic insulin elevations.

## Materials and methods

### Cell culture

RBL2H3 [28] (ATCC CRL-2256) were grown at 37°C in a 5% CO_2_/95% air incubator maintained at 95% humidity. Cells were maintained in Dulbecco’s Modification of Eagle Medium (Mediatech Inc., Herndon, VA) with 10% heat-inactivated fetal bovine serum (FBS, Mediatech) and 2 mM glutamine. Lipid body accumulation was induced by incubating RBL2H3 in 1–10 μg/ml insulin alone where indicated or with 0.25 μM dexamethasone and 0.25 μM isobutylmethylxanthine in the presence of 10% FBS for 2 or 6 d at 37°C, beginning 24 h after seeding.

### Chemicals, reagents, and stimulants

General chemicals include LipidTOX Red Neutral Lipid Stain Reagent (LipidTox-NR, H34476), 10-nonyl bromide acridine orange (NAO, A-1372), from Molecular Probes (Eugene, OR), 4′,6-diamidino-2-phenylindole (DAPI), Oil Red O (ORO) from EMD (Gibbstown, NJ).

### Functional assays

Resolvin, leukotriene and prostaglandin assays were purchased from Cayman Chemical (Ann Arbor, MI). Assays were developed according to the manufacturer’s instructions, using cell pellets or supernatants derived from 2.5 million RBL2H3 per assay point, performed in triplicate. Mast cell degranulation was assayed as follows: RBL2H3 were plated in cluster plates at 5 × 10 [4] cells/well. Monolayers were washed and incubated in 200 µl Tyrode’s buffer before stimulating as described. After 45 min at 37°C, 25 µl supernatant was removed, clarified by microcentrifugation, and transferred to a 96 well plate containing 100 µl per well 1 mM *p*-N-acetyl glucosamine (Sigma) in 0.05 M citrate buffer pH 4.5. After 1 h at 37°C reactions were quenched by the addition of 100 µl per well 0.2 M glycine, pH 9.0. Beta-hexosaminidase levels were read as OD at 405 nm. Results are shown as the mean ± standard deviation.

### Staining

RBL2H3 cells were seeded on glass coverslips, fixed with 0.4% (w/v) paraformaldehyde (1 h, room temperature [RT]), washed twice with 1X PBS (Phosphate Buffer Saline) and stained with various lipid dyes. Staining with Oil Red O (0.35% in 6:4 EtOH:water, 15 min, RT), DAPI (2.5 μM, 5 min), NAO (200 nM, 1 h), LipidTox-NR (4 μM, 30 min).

### Imaging

Bright field and fluorescence imaging of cells in MatTek dishes (50,000 cells per cm^2^) were performed on a Nikon Ti Eclipse C1 epi-fluorescence and confocal microscopy system, equipped with heated stage. Available laser lines in FITC, TxRed and Cy5 were supplied by a 488 nm 10 mW solid-state laser, a 561 nm 10 mW diode pump solid state laser and a 638 nm 10 mW modulated diode laser. Z stack size was 15 microns. Each z disc (optical section) was 150 nm. Pinhole size for all images was 60 microns. Images were analysed in NIS Elements (Nikon, Melville, NY).

### Lipidomic analysis

The lipids were extracted by the method of Folch et al. using chloroform:methanol (2:1 v/v). For the separation of neutral lipid classes (FFA, triacyl glycerols (TAG), diacylglycerol (DAG), free cholesterol (FC), cholesterol ethers (CE)), a solvent system consisting of petroleum ether/diethyl ether/acetic acid (80:20:1, by vol) was employed. Individual phospholipid classes within each extract are separated by liquid chromatography (Agilent Technologies model 1100 Series). Each lipid class is transesterified in 1% sulfuric acid in methanol in a sealed vial under a nitrogen atmosphere at 100°C for 45 min. The resulting fatty acid methyl esters are extracted from the mixture with hexane containing 0.05% butylated hydroxytoluene and prepared for gas chromatography by sealing the hexane extracts under nitrogen. Fatty acid methyl esters are separated and quantified by capillary gas chromatography (Agilent Technologies model 6890) equipped with a 30 m DB 88 capillary column (Agilent Technologies) and a flame ionization detector. Whole cell lipidomic analysis was performed in collaboration with Metabolon, Inc.

### Lysis and western blot

Cells were pelleted (2000 x *g*, 2 min) and washed once in ice-cold PBS. For total lysates, 1 × 10^7^ cells were lysed (ice, 30 min) in 350 μl lysis buffer (50 mM HEPES [pH 7.4], 250 mM NaCl, 20 mM NaF, 10 mM iodoacetamide, 0.5% (w/v) Triton X-100, 1 mM phenylmethyl-sulphonylfluoride, 500 mg aprotinin/ml, 1.0 mg leupeptin/ml and 2.0 mg chymostatin/ml). Lysates were clarified (17,000 x *g*, 20 min). For preparation of total protein, lysates were acetone precipitated (1.4 vol acetone for 1 h at −20°C, followed by 10,000 x *g* for 5 min). Protein samples were resolved by 10% SDS-PAGE under reducing conditions. Resolved proteins were electrotransferred to PVDF membranes in 192 mM glycine/25 mM Tris (pH 8.8) solutions. For Western blotting, membranes were blocked using 5% non-fat milk in PBS for 1 h at RT. Primary antibodies were dissolved in PBS/0.05% Tween-20/0.05% NaN_3_ and incubated with the membranes for 16 h at 4°C. After washing the membranes four times with PBS/0.1% Tween-20 (5 min/wash), developing antibodies (anti-rabbit or anti-mouse IgGs conjugated to horseradish peroxidase diluted to 0.1 μg/ml in PBS/0.05% Tween-20; Amersham) were placed with the membranes for 45 min at RT. Washes were then performed again, and the adherent secondary antibody then visualized using enhanced chemiluminescence solution (Amersham) and exposure of the membrane to BioMax film (Kodak, Rochester, NY). Films were then scanned at >600 dpi, and quantification of the band intensities performed using Image J (NIH, Bethesda, MD).

### Mitochondrial staining

Mitotracker (Molecular Probes) was used at 1μM for 15 min at 37°C.

### Statistical analysis

Results are shown as mean ± SD or standard error. Statistical significance was determined based on analysis of variance (ANOVA; GraphPad Prism 6 v6.02; San Diego, CA). Adjacent to datapoints in the respective graphs, significant differences were recorded as *p < 0.05, **p < 0.01, or ***p < 0.001. Data shown are all based on an *n* of at least three experiments.

## Results

### Chronic insulin exposure results in lipid body accumulation in RBL2H3

**Figure 1.**
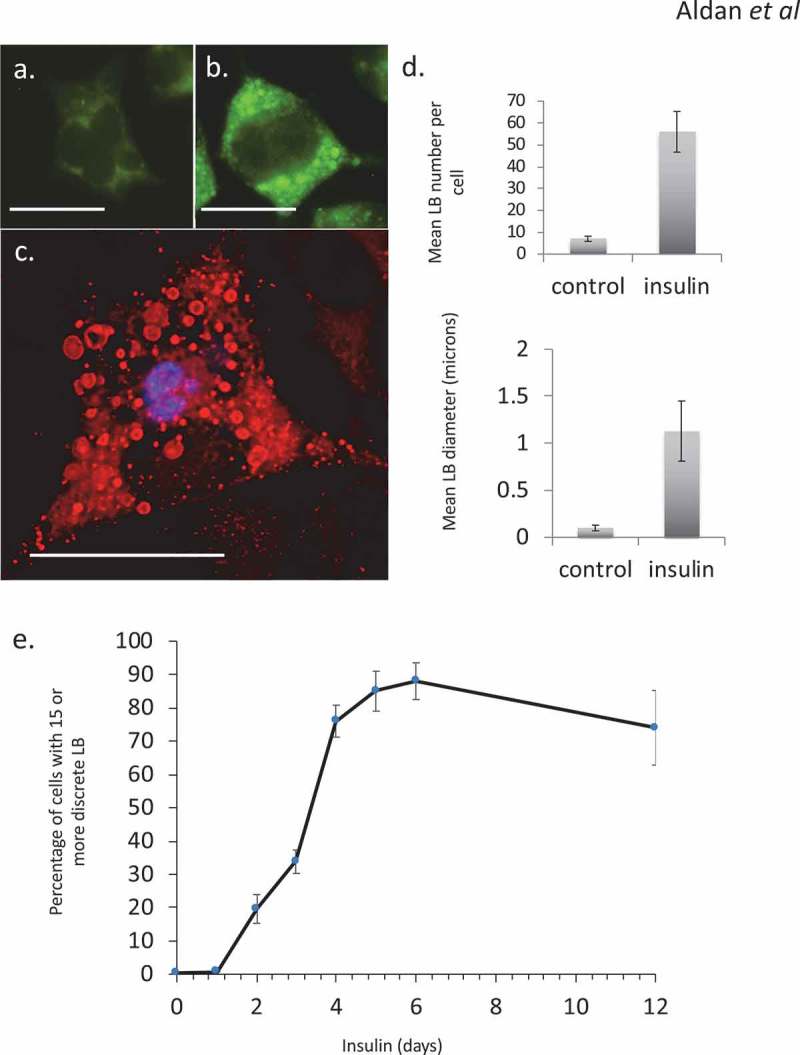
Steatosis in RBL2H3 induced by Insulin. (a–c). RBL2H3 control (a) or 6 day insulin-stimulated (b, c) stained with either LipidTox Green (488nm, A, B) or Oil Red O (c) and imaged using confocal microscopy. Scale bar is 10 microns. (d). *Upper panel*. Mean discrete lipid bodies per cell counted using thresholded Region of Interest (ROI) analysis (Nikon NIS Elements) across 100 control and 100 6 d insulin treated cells. *Lower panel*. Average LB diameter (in microns) measured for each LB. (e). Time course of lipid body formation in RBL2H3 in response to insulin treatment.

Hyper-insulinemia was mimicked *in vitro* using chronic exposure to elevated insulin levels. Hyper-insulinemia in either escalated dose schedules (not shown) or single-sustained doses resulted in an altered phenotype in RBL2H3. As we have previously described, this phenotype includes the steatotic accumulation of lipid bodies [18,29,30] (Figure 1(a–d), associated with overall gains in lipid content. In the populations of cells used here, at least 80% of the cells were confirmed (by Oil Red O staining and microscopy) to have gained significant lipid body accumulation and exhibit a ‘steatotic’ staining pattern by the 6-d insulin exposure timepoint (Figure 1(e)). These data are consistent with both our prior studies and observations from adipocyte and immunocytes systems that see lipid body accumulation and overall gains in lipid content in response to insulin, nutrient excess and infection, respectively [6–9,31–33].

### Comparison of fatty acid profiles in major lipid classes between control and insulin-treated cells

We used a lipidomic approach to obtain an overview of the fatty acid profile of nine major lipid classes in insulin-treated and control RBL2H3. Figure 2(a) presents a profile of percentage changes in individual fatty acid levels across nine major lipid classes (cholesterol ethers (CE), (DAG), free fatty acids (FFA), (TAG), cardiolipins (CL), lysophosphatidylcholine (LysoPC), (PC), (PE) and (PS)). The analysis included saturated fatty acids (14:0, 16:0, 18:0. 20:0 and 22:0), single-unsaturated fatty acids (14:1n5, 16:1n7, 18:1n7, 18:1n9, 20:1n9 and 22:1n9), Omega-3 (n-3) fatty acids (18:3n3, 18:4n3, 20:3n3, 20:4n3, 20:5n3, 22:5n3, 22:6n3) and Omega-6 (n-6) fatty acids (18:2n6, 18:3n6, 20:2n6, 20:3n6, 20:4n6, 22:2n6, 22:4n6, 22:5n6). In this Figure, the size, location and colour of the data points relate to the lipid class (colour) and the degree of change in abundance relative to control samples from non-insulin-treated cells (size of marker and distance from axis). By visual inspection we can see that areas of the most marked fold changes in lipid abundance occur in the CE, DAG, TAG and FFA classes, in lipids of chain length 18–22, unsaturated, and within the n-6 and n-3 fatty acid families. The overall trend is towards gains in lipid abundance. The few areas of downregulation seem concentrated in the saturated and single unsaturated FA species, although marked loss in the DAG form of 22:4n6 (*all-cis*-7,10,13,16-docosatetraenoic acid) and 18:4 n3 (*all-cis*-6,9,12,15,-octadecatetraenoic acid), and two n-3 and n-6 forms of cardiolipin are apparent. PC, PS and PE pools are regulated across lipid species, as is the cardiolipin (CL) population. Figure 2(b–d) shows the proportional representation of each of the indicated lipid class in cells treated with vehicle or 6-d insulin. We note shifts from PC to PE and PS in the phospholipid classes. Figure 3 evaluates saturation trends between control and insulin-treated cells. Figures 3(a and c) present absolute (nmol per billion cells) measurements of lipid classes in the indicated levels of saturation. For comparison, Figures 3(b and d) present a normalized view of the same changes, allowing us to see proportional expansion or contraction in degrees of saturation for each lipid class. In Figure 3(e–m) we use the same normalization approach to examine insulin-induced shifts in abundance by individual lipid and by class. These normalized presentations allow us to assess remodelling between lipid classes and within individual lipid species in response to 6-d insulin exposure.

**Figure 2.**
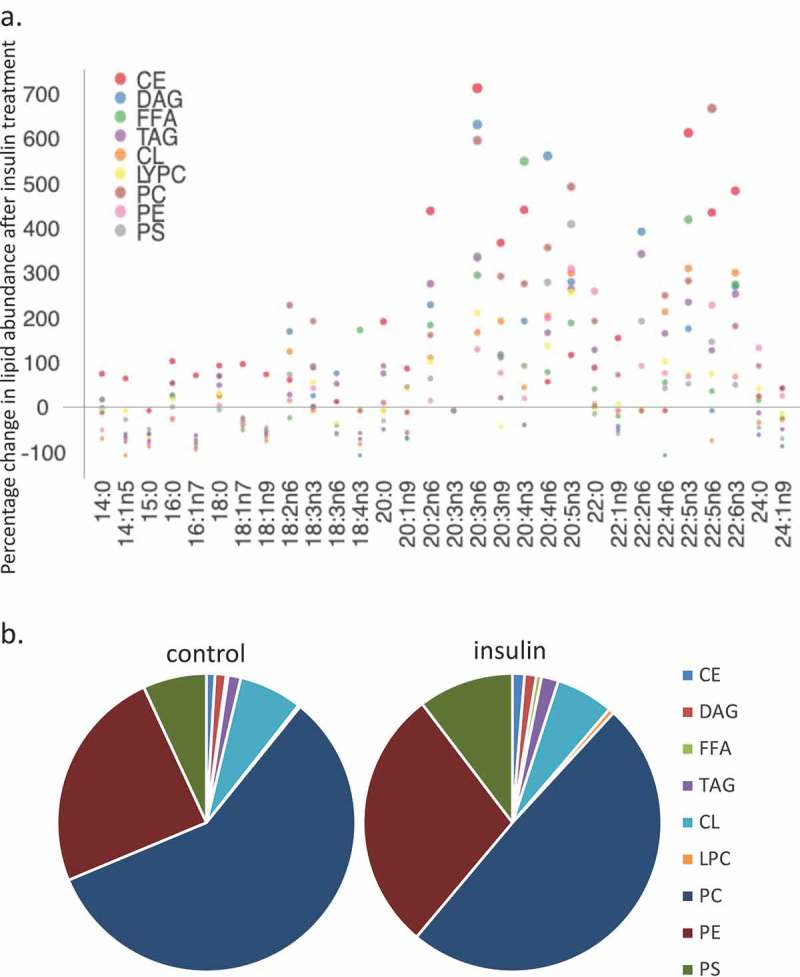
Whole cell lipidome of control and Lipid Body-rich RBL2H3. RBL2H3 were grown for 6d with insulin-FDI. Individual major lipid species were separated by high performance liquid chromatography (HPLC) and fatty acid methyl esters (FAME) from each class were produced and subsequently analyzed by GC/MS. A. Percentage changes in phospholipids, acylglycerols, and free fatty acids as constituents of mast cell lipid bodies. The change is a measure of insulin treated mast cell values minus control cell values divided by the control cell values and multiplied by 100 during the same experimental period. Each color represents a measured species: CE (cholesterol-fatty acid ester), DAG (diacylglycerol), FFA (free fatty acids), TAG (triacylglycerol), CL (cardiolipin), LYPC (lysophosphatidylcholine), PC (phosphatidylcholine), PE (phosphatidylethanolamine), PS (phosphatidylserine). The marker size and position correspond to the percentage change of that lipid, with larger markers and distance from the horizontal axis representing more significant differences between control and insulin-treated samples. B. Proportional representation (by percentage) of lipid classes in control and insulin (6d) treated RBL2H3. C, D. Insulin effects on lipid levels (nmol per billion cells) in the indicated classes. Data are presented on two graphs due to differences in y-axis scaling.

**Figure 2.**
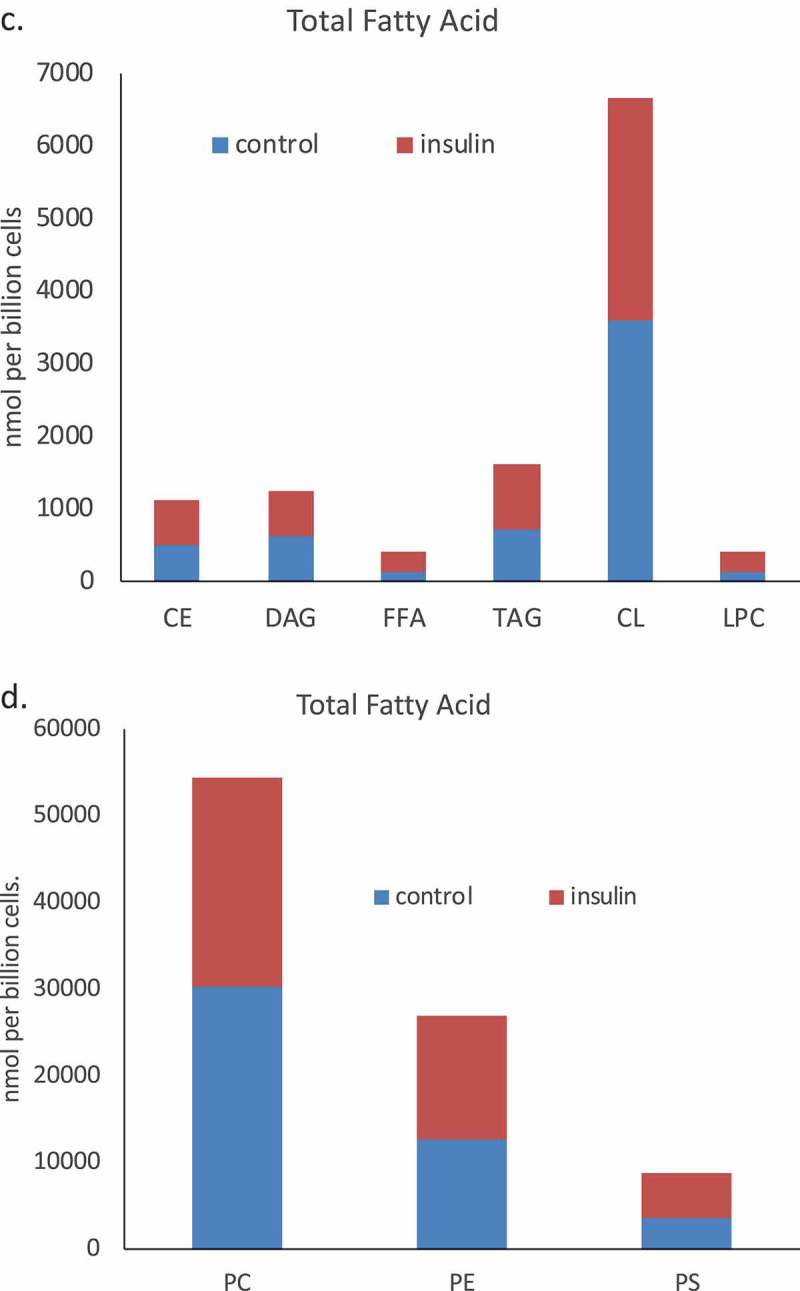
Continued.

**Figure 3.**
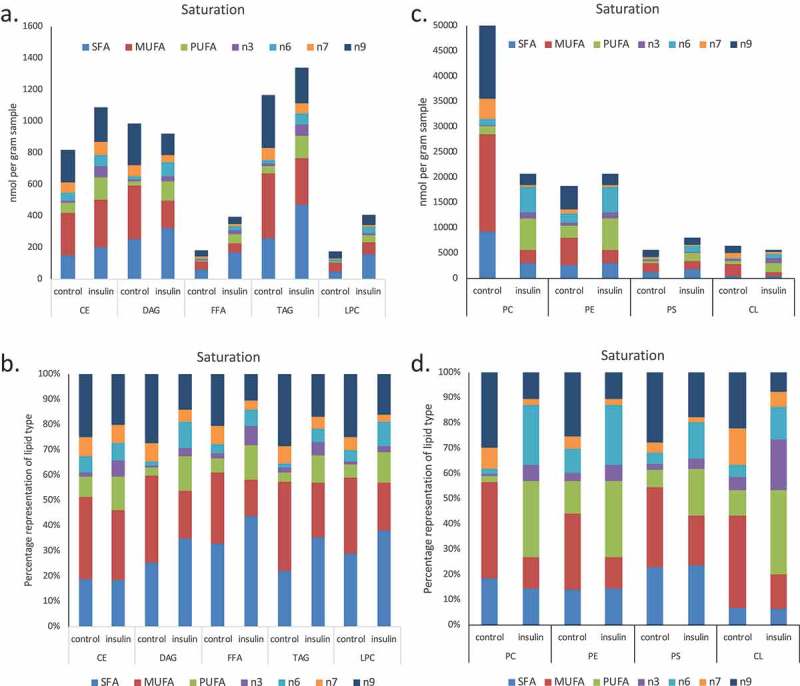
(a–d). Saturation analysis of lipids in RBL2H3 in the absence and presence of chronic insulin exposure. Lipid classes were analysed for the presence of Saturated Fatty Acids (SFA), Mono-unsaturated FA (MUFA), Poly-unsaturated FA (PUFA) and omega n 3, 6, 7, and 9 lipids. Data in A and C present absolute lipid levels (nmol per billion cells). Data in B and D show normalized data to assess proportional representation of SFA, MUFA, etc., in each class in the absence and presence of insulin. Aggregated lipid groups (e.g. PUFA) are shown alongside disaggregated categories (e.g. n3 alone, which is a subset of PUFA). E-M. Normalized (each to 100%) proportion of each lipid species by class in the absence and presence of chronic insulin exposure.

**Figure 3.**
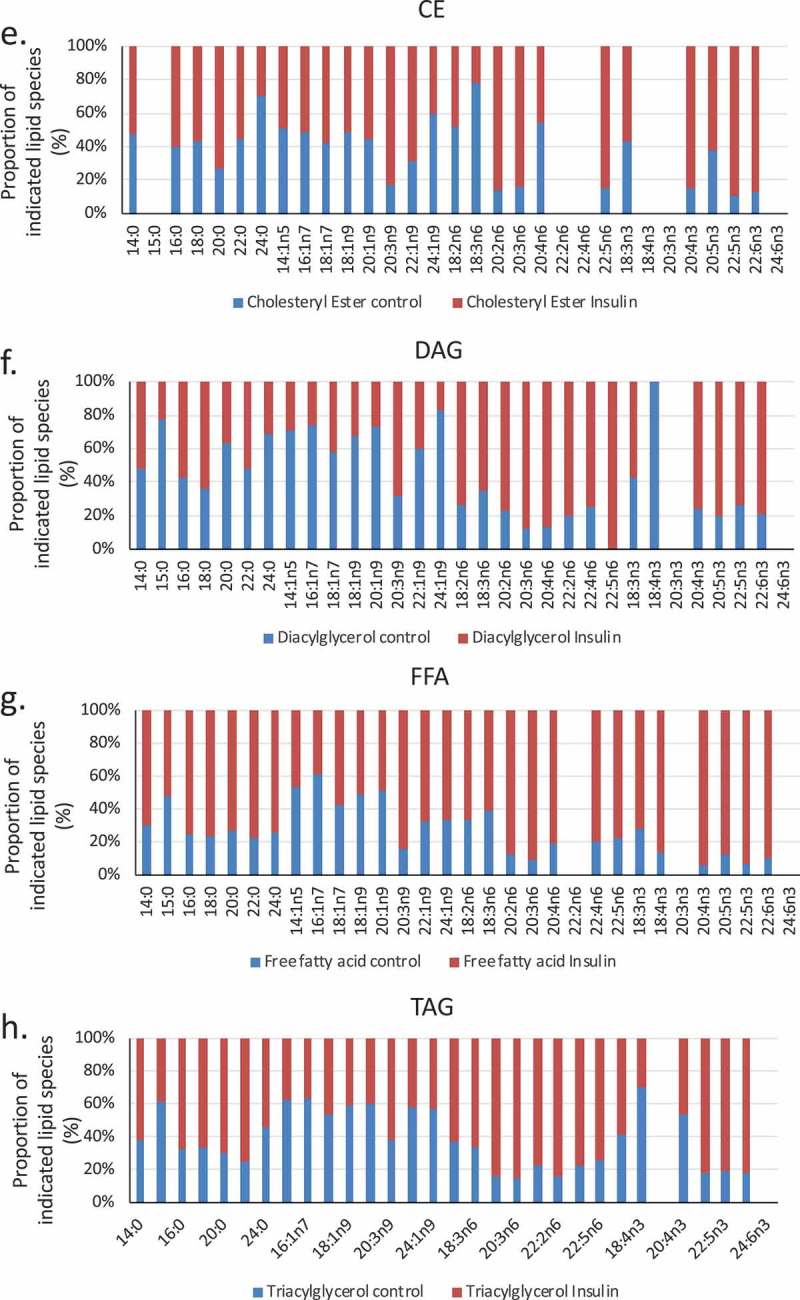
Continued.

**Figure 3.**
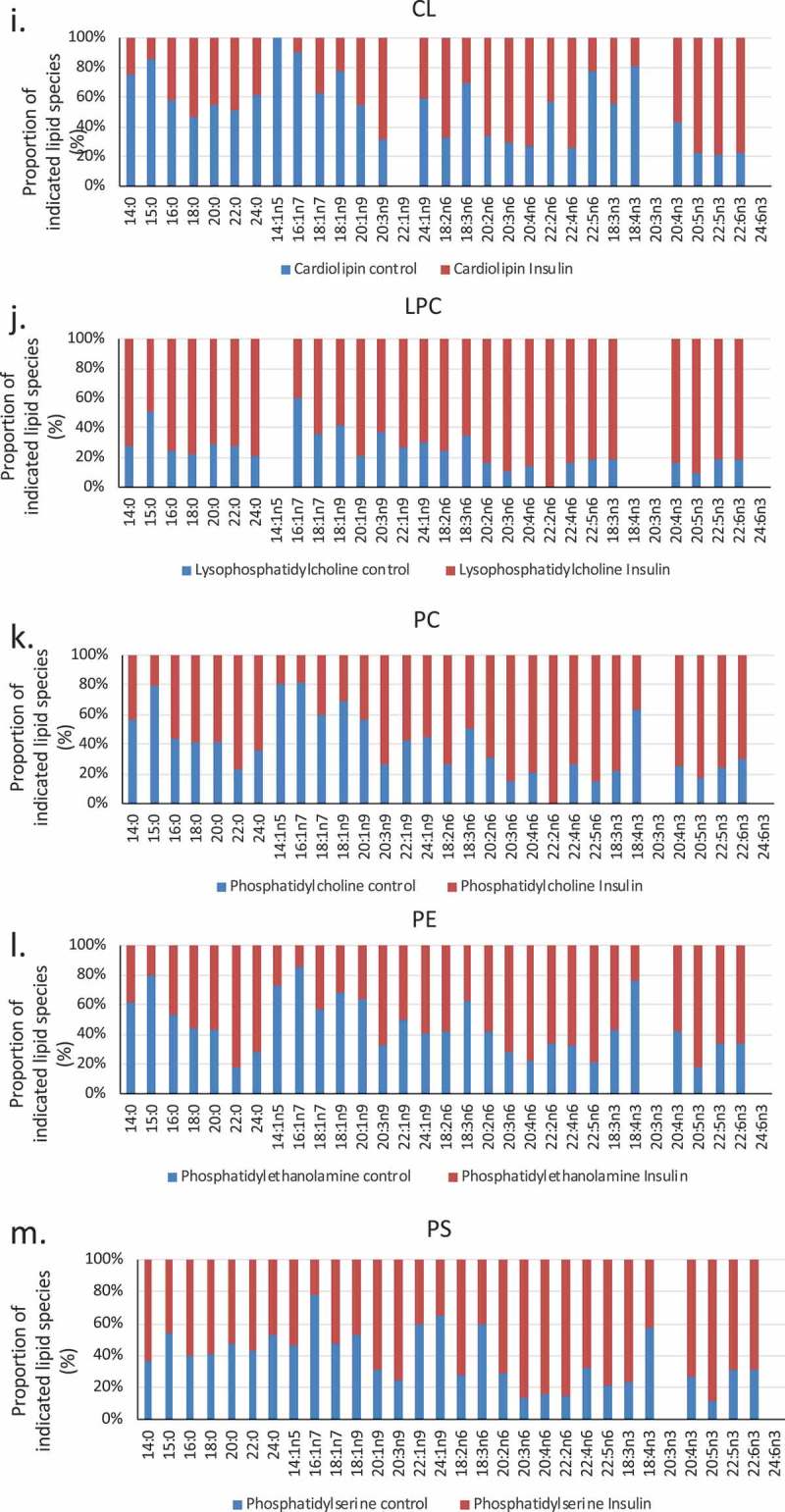
Continued.

### Remodelling of AA, DHA and EPA pools in insulin-treated cells

**Figure 4.**
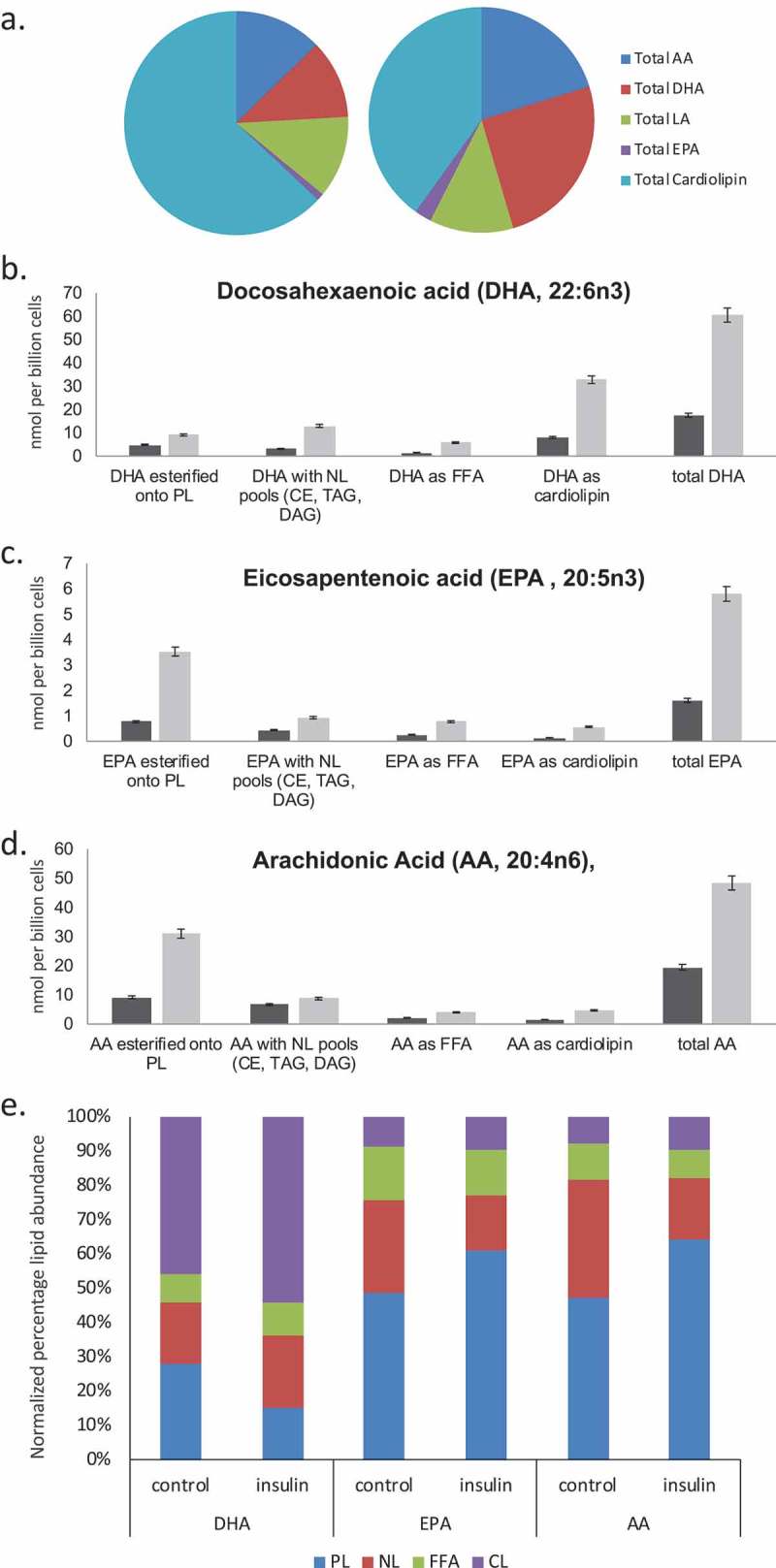
EPA, DHA, AA and Cardiolipin pools are remodelled during chronic insulin exposure in RBL2H3. (a). Pie chart overview of proportions (%) of lipids in each category. (b–d). Absolute levels (nmol per billion cells) of DHA, EPA, AA and CL species localized within specific cellular pools (esterified onto phospholipids (PL), in neutral lipid (NL) pools, as Free Fatty Acids (FFA) or as cardiolipins (CL). E. Normalized percentage abundance of each lipid species by pool in the absence and presence of chronic insulin exposure.

The location of fatty acids within specific lipid pools is of functional significance for the cell. Whether fatty acids such as Arachidonic Acid (AA, 20:4n6), eicosapentenoic acid (EPA, 20:5n3) and Docosahexaenoic acid (DHA, 22:6n3) are in a free fatty acid pool, are esterified onto membrane phospholipids, or are associated with neutral lipid pools changes their ability to be accessed as precursors for the production of bioactive messengers. Organellar locations, for example defined by association with a primarily mitochondrial lipid such as cardiolipin, are also important determinants of lipid availability for signalling and metabolic purposes. We assessed the degree to which insulin-induced remodelling between lipid pools and locations in RBL2H3. Figure 4(a–e) shows the effect on insulin on the distribution of AA, DHA, and EPA between various pools. We assessed the percentages of the total lipid-associated esterified onto phospholipid, associated with neutral lipid pools, present as free fatty acids or associated with cardiolipin (shown in Figure 4(a)). Figure 4(b–d) shows in absolute quantities, the changes in the total DHA, EPA and AA and the changes in the amount associated with phospholipid (PL), neutral lipid (NL) free fatty acid (FFA) and (CL) pools. Figure 4(e) provides a representation normalized to the totals in each category, allowing us to visualize the proportional remodelling that occurs in each pool. We note that DHA remodels towards CL and away from PL. For EPA and AA the NL pool decreases and PL esterification increases.

### Altered functional responses including bioactive lipid release in steatotic mast cells

**Figure 5.**
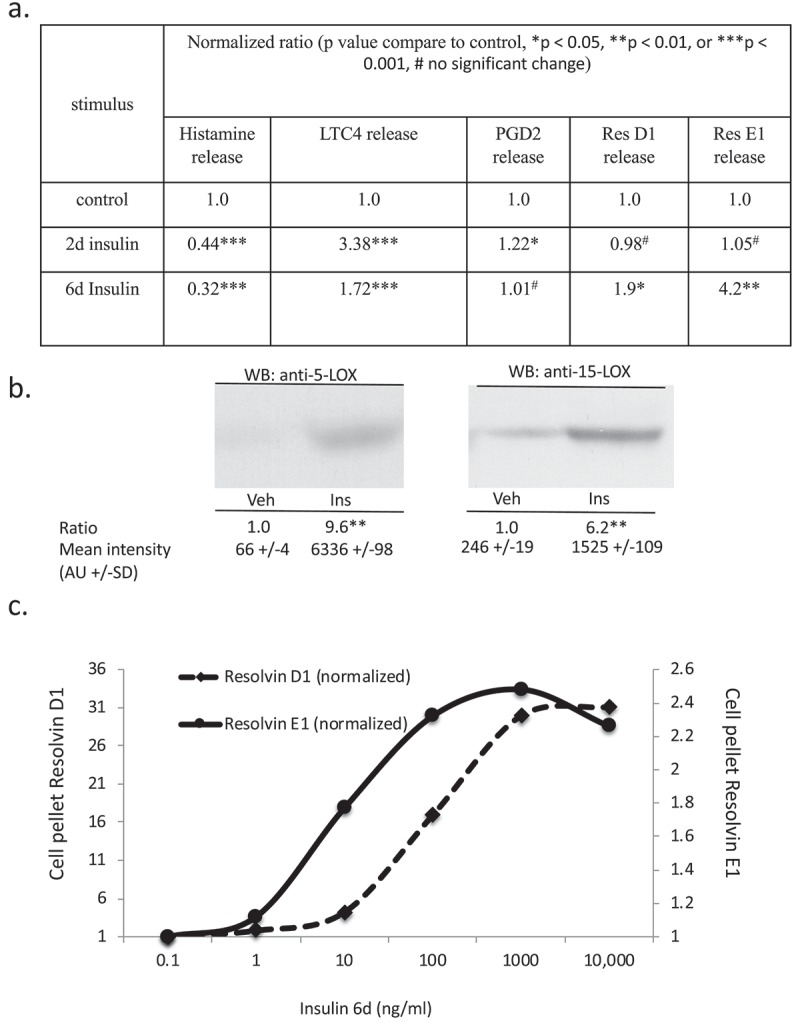
Assessment of histamine and bioactive lipid release by RBL2H3 in the absence and presence of chronic insulin. (a). Summary data for histamine, LTC4, PGD2, Resolvin D1 and E1 release by RBL2H3 in response to FcϵRI stimulation (IgE anti-DNP followed by 200 ng/ml KLH-DNP) after 6 d treatment with either vehicle or insulin (10 μg/ml). (b). Western blots of lysates prepared from control and 6d insulin cells and probed with antibodies to either 5-lipoxygenase or 15-lipoxygenase, as indicated. AU, arbitrary intensity units from densitometry. (c). Dose response of Resolvin D1 and E1 abundance in RBL2H3 following 6 d treatment with the indicated levels of insulin. Resolvin D1: *p* values relative to control for insulin doses: 1ng/ml Ins p > 0.05, 10 ng-10,000ng/ml Ins, p < 0.005. Resolvin E1. 1ng/ml Ins p > 0.05, 10 ng-10,000ng/ml Ins, p < 0.005.

We extended our previous data by evaluating a range of functional responses in control, 2-d and 6-d insulin-exposed cells. Figure 5(a) tabulates the effects of 2 and 6 d insulin exposure on release of histamine, the Series 4 leukotriene LTC4 and the Series 2 prostaglandin PGD2, Resolvin D1 and Resolvin E1 [17]. Figure 5(b) shows the insulin-induced upregulation of key biosynthetic enzymes in the LTC4 and Resolvin synthesis pathways. Figure 5(c) shows a dose response of the impact of insulin on Resolvin D1 and E1 levels.

### Cardiolipin levels and mitochondrial/lipid body complexes are altered in insulin-exposed RBL2H3

**Figure 6.**
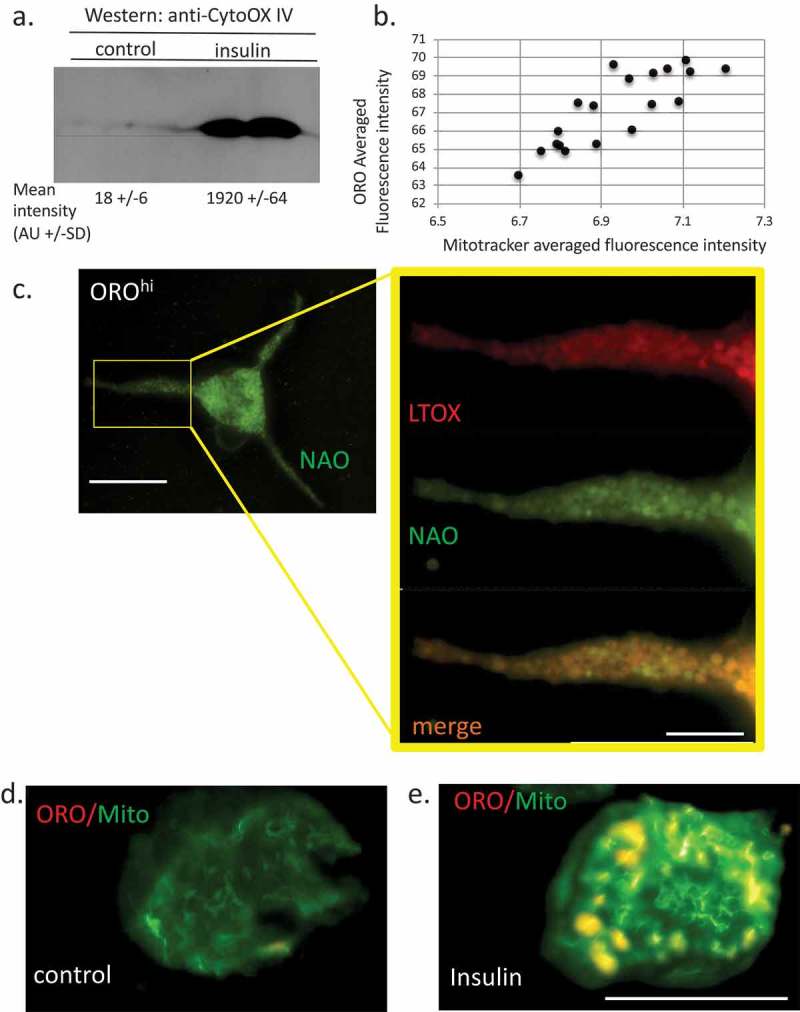
Relationship between lipid bodies, mitochondria and cardiolipins in insulin-treated RBL2H3. (a). Western blot analysis of Cytochrome oxidase IV levels in control and 6 d insulin-treated RBL2H3. AU, arbitrary intensity units from densitometry. (b). Correlation between Mitotracker and ORO staining intensities. Scale bar at left is 10 microns, scale bar at right is 2.5 microns. (c). Co-localization of ORO and NAO staining in RBL2H3 treated for 6d with insulin. (d, e). Matched confocal imaging (all parameters equivalent) of the cell bodies of 2 RBL2H3, control (d) or insulin treated for 6 d (e) stained as indicated with ORO and MitoTracker Green. Scale bars are 10 microns.

Cardiolipin emerged from our analysis as a highly regulated lipid species following chronic insulin exposure. CL was overall increased in abundance by >3 folds and it follows that AA, DHA, EPA and LA all increased their proportional representation in the CL lipid class. Given the mitochondrial function of CL, we would not have expected this lipid to be highly regulated in cells that were simply enriched in lipid bodies. However, there have been several studies showing that LB and mitochondria are physically interacting [34–36] and that there may be functional interactions between the two. In addition, omega-3 and omega-6 FA-induced remodelling in CL [37]. We asked if this correlated with increased mitochondrial load. Figure 6(a) shows that the mitochondrial marker Cytochrome Oxidase IV is upregulated in insulin-treated cells. Mitochondrial abundance is positively correlated with ORO staining intensity (Figure 6(b)). Microscopically, we can image the mitochondrial population under matched conditions in ORO^lo^INS^−^ versus ORO^hi^INS^+^ cells and there is colocalization between both Mitotracker and the CL marker 10-nonyl acridine orange NAO [38] (Figure 6(c), Pearson Correlation Coefficient (PCC) for colocalization (Jacop module, Image J) of 0.85) and ORO/Mitotracker (Figure 6(c), PCC 0.91) and we observe higher levels of Mitotracker fluorescence in the latter at the single cell level (Figure 6(d,e)).

## Discussion

This study characterizes alterations in lipid classes following chronic insulin exposure in a model mast cell line. Our lipidomic evaluation quantified and analyzed 342 distinct lipid molecules consisting of 38 fatty acids in 9 major lipid species. Whole cell lipid extracts from mast cells treated for 6 days with insulin show significant alterations in major lipid species and associated fatty acid composition in respect to fold change and relative abundance. Highly upregulated lipids (by percentage or absolute abundance) include AA, eicosatetraenoic acid (20:4n3), docosapentaenoic acid (22:5n3), docosahexaenoic acid (22:6n3) and di-homo-gamma-linolenic acid (20:3n6). Markedly downregulated lipids (by percentage or absolute abundance) included oleic acid (18:1n9) and palmitoleic acid (16:1n7). We detect significant alterations in the degree of unsaturation in individual fatty acids. Insulin treatment resulted in a significant upregulation of the polyunsaturated fatty acids (PUFAs), specifically the omega-3 and omega-6 PUFAs. PUFAs, in particular the 18–20 carbon long omega-6 PUFAs, constitute the precursor molecules required for AA synthesis. AA is a precursor for production of eicosanoids such as prostaglandins, prostacyclins, thromboxanes and leukotrienes.

We have previously demonstrated that chronic insulin exposure drives the formation of lipid bodies in mast cells, with important collateral effects on mast cell function [39] including a decrease in the intensity of responses such as histamine secretion. In this study, our data suggest that mast cells chronically exposed to insulin *in vitro* also have marked alterations in their lipidomic landscape. Mast cells have been linked to a variety of pathologies defined by an underlying inflammatory component, and the role of mast cell-derived lipid messengers (leukotrienes, prostaglandins, thromboxanes, etc.) in these pathologies is clear [40]. The physiological roles of mast cells in wound healing, regulation of smooth muscle tone and vasoregulation are also dependent upon these lipid messengers [40,41]. Obesity and type 2 diabetes are also inflammatory diseases, and excess adiposity is associated with adiposopathy, where the adipose itself becomes dysfunctional and inflamed [42]. Mast cells have been implicated in adipose inflammation [43], and mast cell stabilization may change outcomes in metabolic syndrome as well as the status of the adipose [44–47]. The finding that insulin changes mast cell phenotype [6–9] has relevance to the status of adipose-relevant mast cells and the mast cell-derived mediators (cytokines, growth factors and bioactive lipids) that affect adipose health.

There are commonalities and differences between the insulin-induced lipid profile changes in mast cells and those previously observed in the obese liver or adipocyte systems that are steatotic [48–50]. Isolated adipocytes from obese tissue, with expanded lipid droplet numbers, show large increases in PUFA and TAG levels [48]. Similarly, adipose tissue as a whole, and the macrophages isolated from it show increased PUFA levels in the obese setting and elevated TAG [50]. TAG are stored in mast cell LB [11]. Our analysis suggests that under the hyperinsulinaemic conditions modelled in our experiments, mast cells show increases in PUFA but marginal increases overall in TAG. The increase in PUFA that we observe, if translated to the mast cells found *in vivo*, could provide a new source for the PUFA that are dramatically elevated in adipose tissue.

As described above, we find the largest lipid increases in response to chronic insulin to occur in the polyunsaturated fatty acid class, which are the precursor molecules responsible for synthesizing a variety of pro-inflammatory and pro-thrombotic agents in the mast cell. More specifically, we observe significant upregulations in the particular fatty acids involved in the biosynthetic pathway of AA, including large increases in the AA molecule (20:4n6) itself. We also found (data not shown) remodelling of AA to different membrane phospholipids (PC, PE, PS) that are consistent with prior studies [19,51,52], and which changes their ability to act as precursors for certain mediators. At least under these experimental conditions, these data indicate that in situations of ectopic lipid deposition caused by chronic exposure to insulin, mast cells may be biased to producing the required lipid-derived precursor molecules necessary and sufficient to drive localized and systemic inflammatory responses. These seem to be supported by the functional assays that we performed to look at leukotriene, prostaglandin and resolvins released after FcϵRI stimulation of control and steatotic cells [53–55]. The concomitant regulation of both pro-inflammatory and pro-resolution mediators is not paradoxical when we consider the stages of inflammation in a situation such as wound healing [41]. The initial establishment of an inflammatory site is guided by pro-inflammatory mediators but the ‘focus’ of the inflammation shifts over time to resolution and tissue repair. The increase in precursors measured lipidomically here appears to translate to real gains in the amounts of fully formed mediators that are released from the cell with impact on both the pro-inflammation and pro-resolution bioactive phases of processes such as wound healing [41]. Moreover, these gains may change outcomes of inflammation reactions in hyperinsulinemic patients [14].
